# Crystal structure of bromido­bis­(naph­thal­en-1-yl)anti­mony(III)

**DOI:** 10.1107/S1600536814020066

**Published:** 2014-09-24

**Authors:** Omar bin Shawkataly, Hafiz Malik Hussien Abdelnasir, Mohd Mustaqim Rosli

**Affiliations:** a Chemical Sciences Programme, School of Distance Education, Universiti Sains Malaysia, 11800 USM, Penang, Malaysia; b X-ray Crystallography Unit, School of Physics, Universiti Sains Malaysia, 11800 USM, Penang, Malaysia

**Keywords:** crystal structure, organo­anti­mony(III) compounds, stibine, hydrogen bonding

## Abstract

In the title compound, [SbBr(C_10_H_7_)_2_], the Sb^III^ atom has a distorted trigonal–pyramidal coordination geometry and the planes of the two naphthalene ring systems make a dihedral angle of 80.26 (18)°. An intra­molecular C—H⋯Br hydrogen bond forms an *S*(5) ring motif. In the crystal, weak C—H⋯Br inter­actions link the mol­ecules into helical chains along the *b-*axis direction.

## Related literature   

For general background to organo­anti­mony(III) compounds and related structures of haloorgano­anti­mony(III) compounds, see: Breunig *et al.* (2008 ▶); Millington & Sowerby (1994 ▶).
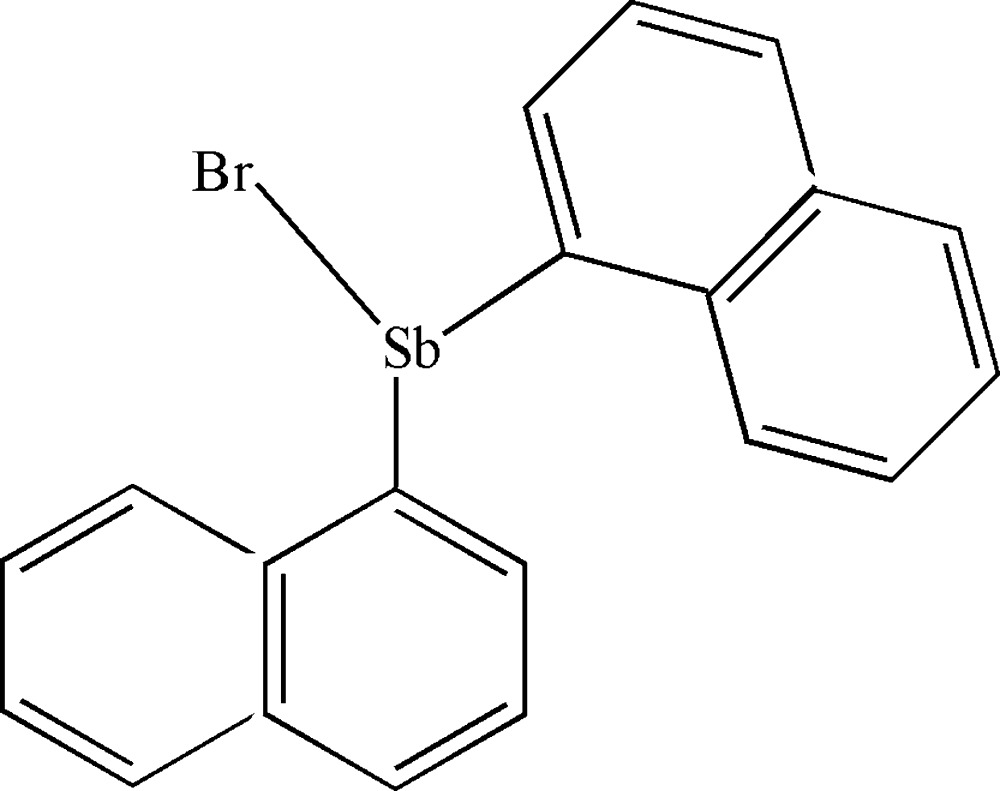


## Experimental   

### Crystal data  

[SbBr(C_10_H_7_)_2_]
*M* *_r_* = 455.97
Monoclinic, 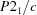

*a* = 12.7371 (3) Å
*b* = 10.9189 (3) Å
*c* = 11.6300 (3) Å
β = 92.661 (1)°
*V* = 1615.70 (7) Å^3^
*Z* = 4
Mo *K*α radiation
μ = 4.17 mm^−1^
*T* = 100 K
0.56 × 0.33 × 0.14 mm

### Data collection  

Bruker SMART APEXII CCD area-detector diffractometer
Absorption correction: multi-scan (*SADABS*; Bruker, 2009 ▶) *T*_min_ = 0.204, *T*_max_ = 0.597
20528 measured reflections
4705 independent reflections
3936 reflections with *I* > 2σ(*I*)
*R*_int_ = 0.032

### Refinement  

*R*[*F* ^2^ > 2σ(*F* ^2^)] = 0.060
*wR*(*F* ^2^) = 0.198
*S* = 1.06
4705 reflections
199 parameters
H-atom parameters constrained
Δρ_max_ = 3.68 e Å^−3^
Δρ_min_ = −3.12 e Å^−3^

### 

Data collection: *APEX2* (Bruker, 2009 ▶); cell refinement: *SAINT* (Bruker, 2009 ▶); data reduction: *SAINT*; program(s) used to solve structure: *SHELXTL* (Sheldrick, 2008 ▶); program(s) used to refine structure: *SHELXTL*; molecular graphics: *SHELXTL*; software used to prepare material for publication: *SHELXTL* and *PLATON* (Spek, 2009 ▶).

## Supplementary Material

Crystal structure: contains datablock(s) I, New_Global_Publ_Block. DOI: 10.1107/S1600536814020066/is5373sup1.cif

Structure factors: contains datablock(s) I. DOI: 10.1107/S1600536814020066/is5373Isup2.hkl

Supporting information file. DOI: 10.1107/S1600536814020066/is5373Isup3.cml

**Fig. 1.**
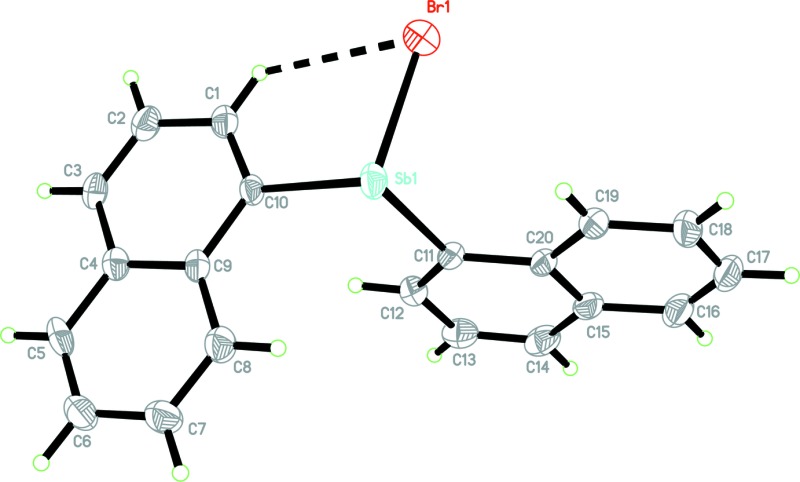
The molecular structure of the title compound, showing 50% probability displacement ellipsoids. The dashed line indicates the C—H···Br hydrogen bond.

. DOI: 10.1107/S1600536814020066/is5373fig1.tif
The mol­ecular structure of the title compound, showing 50% probability displacement ellipsoids. The dashed line indicates the C—H⋯Br hydrogen bond.

**Fig. 2.**
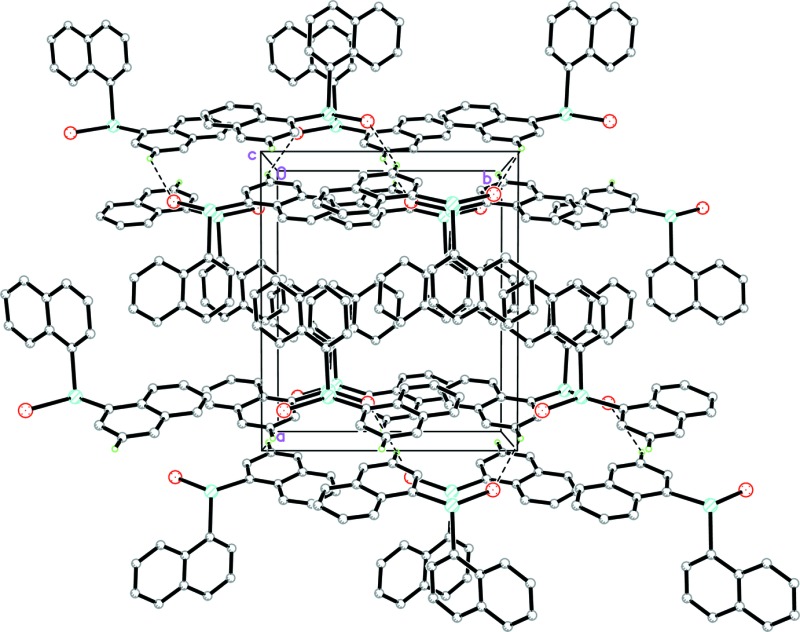
A crystal packing view of the title compound. Dashed lines indicate hydrogen bonds. H atoms not involved in the hydrogen bonds have been omitted for clarity.

. DOI: 10.1107/S1600536814020066/is5373fig2.tif
A crystal packing view of the title compound. Dashed lines indicate hydrogen bonds. H atoms not involved in the hydrogen bonds have been omitted for clarity.

CCDC reference: 1023098

Additional supporting information:  crystallographic information; 3D view; checkCIF report

## Figures and Tables

**Table 1:** Hydrogen-bond geometry (Å, °)

*D*—H⋯*A*	*D*—H	H⋯*A*	*D*⋯*A*	*D*—H⋯*A*
C1—H1*A*⋯Br1	0.95	2.71	3.408 (6)	130
C2—H2*A*⋯Br1^i^	0.95	2.96	3.698 (6)	135

Symmetry code: (i) 
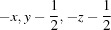
.

## supplementary crystallographic information

### S1. Experimental

All synthetic reactions were performed under dry, oxygen-free dinitrogen atmosphere using standard Schlenk techniques; THF was dried over sodium and distilled from sodium benzophenone ketyl under nitrogen. Antimony trichloride, 1-bromo-naphthaline, and magnesium filing purchased from Sigma Aldrich. The title compound was prepared by adding a solution of antimony trichloride (0.9124 g, 0.0040 mol) in 30 ml THF was added dropwise with stirring to a Grignard mixture of magnesium filings (0.31 g, 0.0129 mol) and 1-bromonaphthaline (1.72 g, 0.0083 mol). The reaction mixture was stirred for 12 h, the solvent was removed in vacuum and the remaining solid was recrystallized from ethanol.

### S2. Refinement

All H atoms were positioned geometrically and refined using a riding model with with C—H = 0.95 Å and *U*_iso_(H) = 1.2*U*_eq_(C).

### Crystal data

**Table:**

[SbBr(C_10_H_7_)_2_]	*F*(000) = 880
*M**_r_* = 455.97	*D*_x_ = 1.875 Mg m^−^^3^
Monoclinic, *P*2_1_/*c*	Mo *K*α radiation, λ = 0.71073 Å
Hall symbol: -P 2ybc	Cell parameters from 9971 reflections
*a* = 12.7371 (3) Å	θ = 2.5–32.2°
*b* = 10.9189 (3) Å	µ = 4.17 mm^−^^1^
*c* = 11.6300 (3) Å	*T* = 100 K
β = 92.661 (1)°	Block, yellow
*V* = 1615.70 (7) Å^3^	0.56 × 0.33 × 0.14 mm
*Z* = 4	

### Data collection

**Table:**

Bruker SMART APEXII CCD area-detector diffractometer	4705 independent reflections
Radiation source: fine-focus sealed tube	3936 reflections with *I* > 2σ(*I*)
Graphite monochromator	*R*_int_ = 0.032
φ and ω scans	θ_max_ = 30.0°, θ_min_ = 2.5°
Absorption correction: multi-scan (*SADABS*; Bruker, 2009)	*h* = −17→17
*T*_min_ = 0.204, *T*_max_ = 0.597	*k* = −15→12
20528 measured reflections	*l* = −16→16

### Refinement

**Table:**

Refinement on *F*^2^	Primary atom site location: structure-invariant direct methods
Least-squares matrix: full	Secondary atom site location: difference Fourier map
*R*[*F*^2^ > 2σ(*F*^2^)] = 0.060	Hydrogen site location: inferred from neighbouring sites
*wR*(*F*^2^) = 0.198	H-atom parameters constrained
*S* = 1.06	*w* = 1/[σ^2^(*F*_o_^2^) + (0.112*P*)^2^ + 12.2014*P*] where *P* = (*F*_o_^2^ + 2*F*_c_^2^)/3
4705 reflections	(Δ/σ)_max_ < 0.001
199 parameters	Δρ_max_ = 3.68 e Å^−^^3^
0 restraints	Δρ_min_ = −3.12 e Å^−^^3^

### Special details

**Table:**

Experimental. The crystal was placed in the cold stream of an Oxford Cryosystems Cobra open-flow nitrogen cryostat operating at 100.0 (1) K.
Geometry. All e.s.d.'s (except the e.s.d. in the dihedral angle between two l.s. planes) are estimated using the full covariance matrix. The cell e.s.d.'s are taken into account individually in the estimation of e.s.d.'s in distances, angles and torsion angles; correlations between e.s.d.'s in cell parameters are only used when they are defined by crystal symmetry. An approximate (isotropic) treatment of cell e.s.d.'s is used for estimating e.s.d.'s involving l.s. planes.
Refinement. Refinement of *F*^2^ against ALL reflections. The weighted *R*-factor *wR* and goodness of fit *S* are based on *F*^2^, conventional *R*-factors *R* are based on *F*, with *F* set to zero for negative *F*^2^. The threshold expression of *F*^2^ > σ(*F*^2^) is used only for calculating *R*-factors(gt) *etc*. and is not relevant to the choice of reflections for refinement. *R*-factors based on *F*^2^ are statistically about twice as large as those based on *F*, and *R*- factors based on ALL data will be even larger.

### Fractional atomic coordinates and isotropic or equivalent isotropic displacement parameters (Å^2^)

**Table:**

	*x*	*y*	*z*	*U*_iso_*/*U*_eq_	
Sb1	0.17759 (3)	0.74179 (3)	0.07848 (3)	0.02490 (15)	
Br1	0.13863 (7)	0.90878 (7)	−0.06577 (8)	0.0494 (3)	
C1	0.0943 (5)	0.6110 (5)	−0.1412 (5)	0.0257 (11)	
H1A	0.0838	0.6928	−0.1672	0.031*	
C2	0.0591 (5)	0.5128 (6)	−0.2125 (5)	0.0292 (12)	
H2A	0.0267	0.5290	−0.2862	0.035*	
C3	0.0717 (5)	0.3958 (6)	−0.1758 (5)	0.0261 (11)	
H3A	0.0482	0.3304	−0.2244	0.031*	
C4	0.1192 (4)	0.3695 (5)	−0.0659 (5)	0.0230 (10)	
C5	0.1307 (5)	0.2481 (5)	−0.0249 (6)	0.0288 (13)	
H5A	0.1061	0.1822	−0.0721	0.035*	
C6	0.1765 (5)	0.2234 (6)	0.0814 (6)	0.0313 (13)	
H6A	0.1831	0.1413	0.1078	0.038*	
C7	0.2140 (6)	0.3218 (6)	0.1516 (6)	0.0326 (13)	
H7A	0.2463	0.3050	0.2252	0.039*	
C8	0.2043 (5)	0.4399 (6)	0.1150 (5)	0.0289 (12)	
H8A	0.2299	0.5044	0.1634	0.035*	
C9	0.1567 (4)	0.4681 (5)	0.0055 (5)	0.0212 (10)	
C10	0.1425 (4)	0.5910 (5)	−0.0364 (5)	0.0206 (10)	
C11	0.3440 (4)	0.7501 (5)	0.0575 (5)	0.0220 (10)	
C12	0.3925 (5)	0.6658 (5)	−0.0089 (5)	0.0244 (10)	
H12A	0.3522	0.6024	−0.0453	0.029*	
C13	0.5023 (5)	0.6718 (6)	−0.0241 (5)	0.0309 (12)	
H13A	0.5354	0.6120	−0.0695	0.037*	
C14	0.5603 (5)	0.7642 (6)	0.0268 (6)	0.0314 (13)	
H14A	0.6339	0.7672	0.0171	0.038*	
C15	0.5130 (5)	0.8555 (6)	0.0936 (5)	0.0278 (12)	
C16	0.5719 (5)	0.9541 (6)	0.1423 (6)	0.0351 (14)	
H16A	0.6452	0.9590	0.1310	0.042*	
C17	0.5250 (6)	1.0422 (7)	0.2053 (6)	0.0424 (18)	
H17A	0.5653	1.1091	0.2354	0.051*	
C18	0.4171 (6)	1.0344 (6)	0.2257 (6)	0.0370 (15)	
H18A	0.3853	1.0948	0.2715	0.044*	
C19	0.3575 (5)	0.9398 (5)	0.1799 (5)	0.0277 (11)	
H19A	0.2847	0.9359	0.1940	0.033*	
C20	0.4029 (4)	0.8482 (5)	0.1120 (5)	0.0233 (10)	

### Atomic displacement parameters (Å^2^)

**Table:**

	*U*^11^	*U*^22^	*U*^33^	*U*^12^	*U*^13^	*U*^23^
Sb1	0.0235 (2)	0.0210 (2)	0.0304 (2)	−0.00274 (13)	0.00205 (15)	−0.00743 (13)
Br1	0.0450 (4)	0.0284 (4)	0.0729 (6)	0.0000 (3)	−0.0185 (4)	0.0040 (3)
C1	0.025 (3)	0.023 (3)	0.028 (3)	−0.006 (2)	−0.001 (2)	0.001 (2)
C2	0.028 (3)	0.033 (3)	0.026 (3)	−0.005 (2)	−0.004 (2)	−0.004 (2)
C3	0.027 (3)	0.027 (3)	0.025 (2)	−0.008 (2)	−0.001 (2)	−0.007 (2)
C4	0.023 (2)	0.021 (2)	0.026 (2)	−0.0027 (19)	0.0031 (19)	−0.0039 (19)
C5	0.032 (3)	0.015 (2)	0.039 (3)	−0.005 (2)	0.009 (3)	−0.004 (2)
C6	0.031 (3)	0.021 (3)	0.042 (4)	0.000 (2)	0.006 (3)	0.005 (2)
C7	0.041 (3)	0.028 (3)	0.028 (3)	0.001 (3)	−0.003 (2)	0.006 (2)
C8	0.034 (3)	0.025 (3)	0.027 (3)	−0.004 (2)	−0.003 (2)	−0.003 (2)
C9	0.020 (2)	0.019 (2)	0.025 (2)	−0.0038 (18)	0.0020 (18)	−0.0027 (19)
C10	0.019 (2)	0.015 (2)	0.028 (3)	−0.0010 (17)	0.0013 (18)	−0.0054 (18)
C11	0.018 (2)	0.022 (3)	0.026 (2)	−0.0024 (18)	−0.0040 (19)	0.0023 (18)
C12	0.025 (3)	0.021 (2)	0.027 (3)	0.000 (2)	0.000 (2)	−0.0011 (19)
C13	0.027 (3)	0.034 (3)	0.032 (3)	0.005 (2)	0.002 (2)	0.003 (2)
C14	0.019 (3)	0.042 (4)	0.033 (3)	−0.001 (2)	0.000 (2)	0.007 (2)
C15	0.027 (3)	0.028 (3)	0.027 (3)	−0.006 (2)	−0.009 (2)	0.010 (2)
C16	0.033 (3)	0.035 (3)	0.036 (3)	−0.013 (3)	−0.012 (2)	0.010 (3)
C17	0.056 (4)	0.031 (3)	0.037 (3)	−0.018 (3)	−0.026 (3)	0.011 (3)
C18	0.053 (4)	0.024 (3)	0.033 (3)	−0.004 (3)	−0.017 (3)	−0.003 (2)
C19	0.034 (3)	0.022 (3)	0.026 (3)	−0.001 (2)	−0.010 (2)	0.000 (2)
C20	0.026 (3)	0.021 (2)	0.022 (2)	−0.004 (2)	−0.0084 (19)	0.0029 (19)

### Geometric parameters (Å, º)

**Table:**

Sb1—C11	2.146 (6)	C9—C10	1.436 (7)
Sb1—C10	2.155 (5)	C11—C12	1.367 (8)
Sb1—Br1	2.5116 (9)	C11—C20	1.438 (7)
C1—C10	1.357 (8)	C12—C13	1.420 (9)
C1—C2	1.416 (8)	C12—H12A	0.9500
C1—H1A	0.9500	C13—C14	1.369 (10)
C2—C3	1.354 (9)	C13—H13A	0.9500
C2—H2A	0.9500	C14—C15	1.416 (10)
C3—C4	1.418 (8)	C14—H14A	0.9500
C3—H3A	0.9500	C15—C16	1.416 (8)
C4—C5	1.414 (8)	C15—C20	1.431 (8)
C4—C9	1.429 (7)	C16—C17	1.363 (12)
C5—C6	1.368 (10)	C16—H16A	0.9500
C5—H5A	0.9500	C17—C18	1.409 (12)
C6—C7	1.419 (10)	C17—H17A	0.9500
C6—H6A	0.9500	C18—C19	1.374 (8)
C7—C8	1.362 (9)	C18—H18A	0.9500
C7—H7A	0.9500	C19—C20	1.414 (9)
C8—C9	1.418 (8)	C19—H19A	0.9500
C8—H8A	0.9500		
			
C11—Sb1—C10	98.0 (2)	C9—C10—Sb1	119.0 (4)
C11—Sb1—Br1	93.38 (15)	C12—C11—C20	120.7 (5)
C10—Sb1—Br1	96.42 (15)	C12—C11—Sb1	120.7 (4)
C10—C1—C2	121.4 (5)	C20—C11—Sb1	118.6 (4)
C10—C1—H1A	119.3	C11—C12—C13	120.9 (5)
C2—C1—H1A	119.3	C11—C12—H12A	119.5
C3—C2—C1	120.0 (5)	C13—C12—H12A	119.5
C3—C2—H2A	120.0	C14—C13—C12	119.6 (6)
C1—C2—H2A	120.0	C14—C13—H13A	120.2
C2—C3—C4	121.0 (5)	C12—C13—H13A	120.2
C2—C3—H3A	119.5	C13—C14—C15	121.4 (6)
C4—C3—H3A	119.5	C13—C14—H14A	119.3
C5—C4—C3	121.8 (5)	C15—C14—H14A	119.3
C5—C4—C9	118.9 (5)	C14—C15—C16	121.6 (6)
C3—C4—C9	119.2 (5)	C14—C15—C20	119.2 (5)
C6—C5—C4	121.5 (6)	C16—C15—C20	119.2 (6)
C6—C5—H5A	119.2	C17—C16—C15	121.0 (6)
C4—C5—H5A	119.2	C17—C16—H16A	119.5
C5—C6—C7	119.3 (6)	C15—C16—H16A	119.5
C5—C6—H6A	120.4	C16—C17—C18	120.2 (6)
C7—C6—H6A	120.4	C16—C17—H17A	119.9
C8—C7—C6	120.9 (6)	C18—C17—H17A	119.9
C8—C7—H7A	119.6	C19—C18—C17	120.4 (7)
C6—C7—H7A	119.6	C19—C18—H18A	119.8
C7—C8—C9	121.0 (6)	C17—C18—H18A	119.8
C7—C8—H8A	119.5	C18—C19—C20	121.0 (6)
C9—C8—H8A	119.5	C18—C19—H19A	119.5
C8—C9—C4	118.4 (5)	C20—C19—H19A	119.5
C8—C9—C10	123.3 (5)	C19—C20—C15	118.2 (5)
C4—C9—C10	118.3 (5)	C19—C20—C11	123.7 (5)
C1—C10—C9	120.0 (5)	C15—C20—C11	118.1 (5)
C1—C10—Sb1	120.4 (4)		
			
C10—C1—C2—C3	1.2 (9)	C10—Sb1—C11—C12	−3.9 (5)
C1—C2—C3—C4	0.4 (9)	Br1—Sb1—C11—C12	−100.9 (5)
C2—C3—C4—C5	178.4 (6)	C10—Sb1—C11—C20	174.3 (4)
C2—C3—C4—C9	−1.6 (9)	Br1—Sb1—C11—C20	77.4 (4)
C3—C4—C5—C6	−179.9 (6)	C20—C11—C12—C13	1.0 (9)
C9—C4—C5—C6	0.2 (9)	Sb1—C11—C12—C13	179.2 (4)
C4—C5—C6—C7	−0.5 (10)	C11—C12—C13—C14	−1.0 (9)
C5—C6—C7—C8	0.4 (10)	C12—C13—C14—C15	−0.8 (10)
C6—C7—C8—C9	−0.1 (10)	C13—C14—C15—C16	−177.3 (6)
C7—C8—C9—C4	−0.2 (9)	C13—C14—C15—C20	2.5 (9)
C7—C8—C9—C10	178.7 (6)	C14—C15—C16—C17	179.2 (6)
C5—C4—C9—C8	0.2 (8)	C20—C15—C16—C17	−0.6 (9)
C3—C4—C9—C8	−179.8 (5)	C15—C16—C17—C18	1.9 (10)
C5—C4—C9—C10	−178.7 (5)	C16—C17—C18—C19	−1.8 (10)
C3—C4—C9—C10	1.3 (8)	C17—C18—C19—C20	0.4 (9)
C2—C1—C10—C9	−1.5 (9)	C18—C19—C20—C15	0.9 (8)
C2—C1—C10—Sb1	−172.3 (4)	C18—C19—C20—C11	−177.2 (6)
C8—C9—C10—C1	−178.6 (6)	C14—C15—C20—C19	179.5 (5)
C4—C9—C10—C1	0.2 (8)	C16—C15—C20—C19	−0.8 (8)
C8—C9—C10—Sb1	−7.6 (7)	C14—C15—C20—C11	−2.4 (8)
C4—C9—C10—Sb1	171.2 (4)	C16—C15—C20—C11	177.4 (5)
C11—Sb1—C10—C1	−105.6 (5)	C12—C11—C20—C19	178.7 (5)
Br1—Sb1—C10—C1	−11.2 (5)	Sb1—C11—C20—C19	0.5 (7)
C11—Sb1—C10—C9	83.5 (4)	C12—C11—C20—C15	0.7 (8)
Br1—Sb1—C10—C9	177.8 (4)	Sb1—C11—C20—C15	−177.6 (4)

### Hydrogen-bond geometry (Å, º)

**Table:**

*D*—H···*A*	*D*—H	H···*A*	*D*···*A*	*D*—H···*A*
C1—H1*A*···Br1	0.95	2.71	3.408 (6)	130
C2—H2*A*···Br1^i^	0.95	2.96	3.698 (6)	135

Symmetry code: (i) −*x*, *y*−1/2, −*z*−1/2.
