# Supplementary figures and images for: Crystal structure of bromido­bis­(naph­thal­en-1-yl)anti­mony(III)
Source: Acta Crystallogr Sect E Struct Rep Online. 2014 Sep 24;70(Pt 10):m351. doi: 10.1107/S1600536814020066 (PMC4257233; doi:10.1107/S1600536814020066)

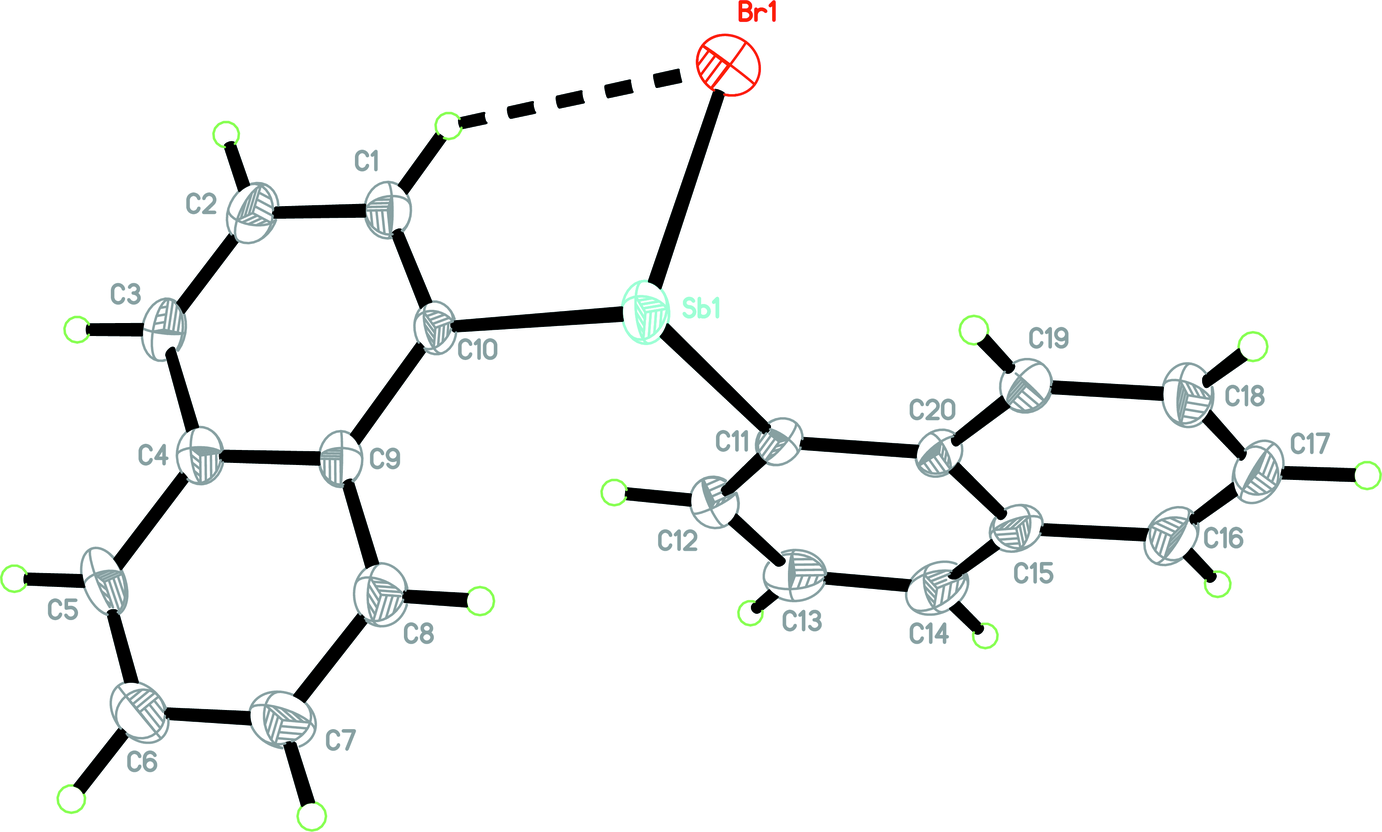

Supplement: Supplementary file 4 [file e-70-0m351-fig1.tif]

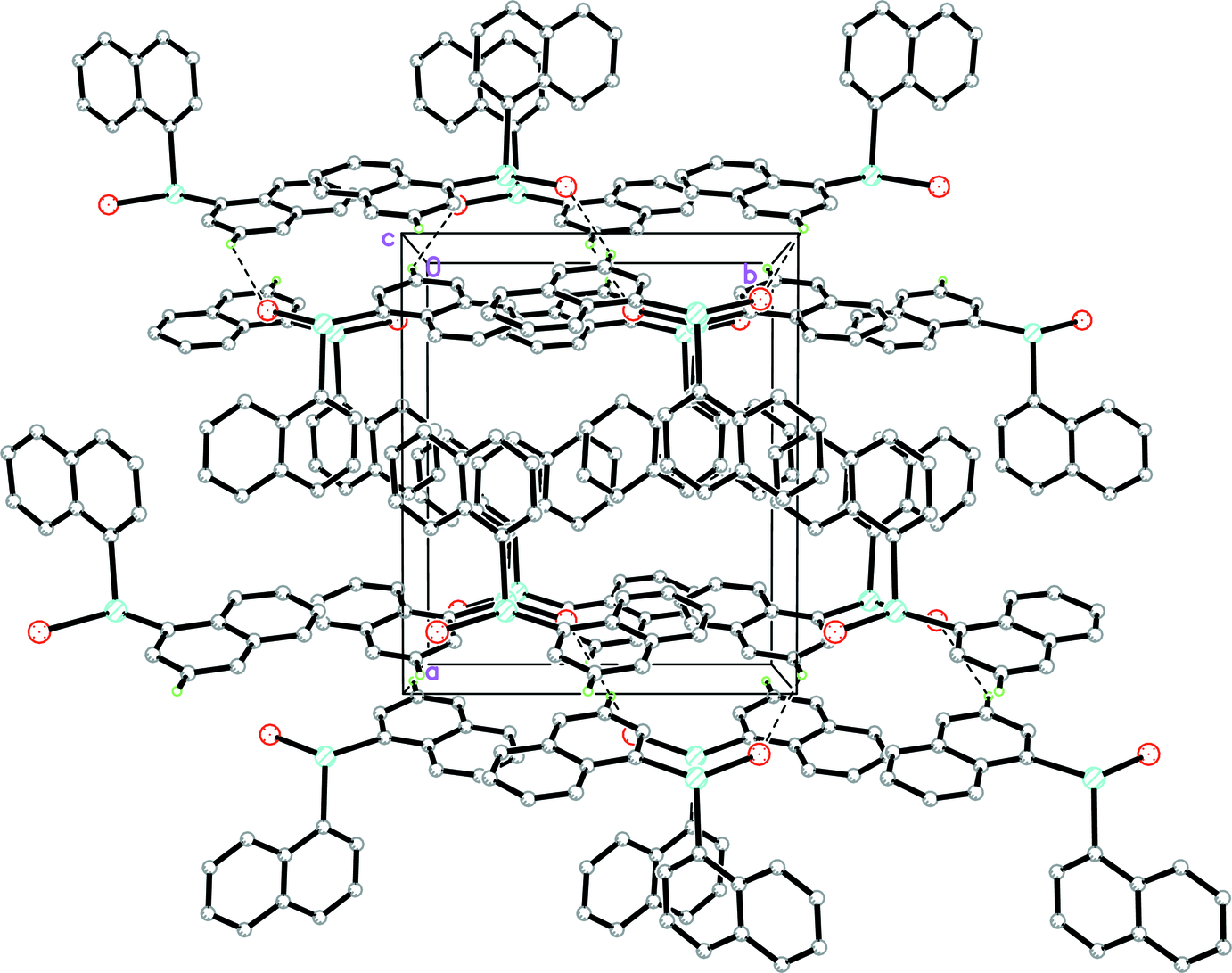

Supplement: Supplementary file 5 [file e-70-0m351-fig2.tif]
